# Sequestered Lumbar Disc Herniation Mimicking a Spinal Tumor: A Case Report

**DOI:** 10.7759/cureus.116552

**Published:** 2026-09-19

**Authors:** Ghaith Khader, Ivan Momotiuk, Mohamad-Khir Alkorayshi, Ralf Buhl

**Affiliations:** 1 Department of Neurological Surgery, Städtisches Klinikum Solingen, Solingen, DEU; 2 Department of Neuropathology, Universitätsklinikum Düsseldorf, Düsseldorf, DEU

**Keywords:** disc sequestration, extradural lesion, lumbar disc herniation, magnetic resonance imaging, spinal tumor

## Abstract

Sequestered lumbar disc fragments are a well-recognized cause of radiculopathy; however, when disc material demonstrates contrast enhancement, it may closely resemble an intraspinal tumor on magnetic resonance imaging (MRI), leading to diagnostic uncertainty, particularly in patients with a history of malignancy. We present the case of a 75-year-old man with a history of sigmoid and prostate carcinomas who presented with persistent low back pain and right-sided radicular symptoms for two months. Neurological examination revealed no motor deficit, with preserved strength of 5/5, although intermittent paresthesia and hyperesthesia and a positive right straight-leg-raise test were present. MRI demonstrated an irregularly shaped, contrast-enhancing lateral extradural lesion at L4/L5, measuring approximately 29 × 13 × 10 mm and maintaining contact with both the anterior and posterior epidural spaces. Associated degenerative changes included loss of disc height and facet arthropathy without relevant central spinal canal stenosis.

Given the patient's oncological history and the radiological appearance, an extradural spinal neoplasm was suspected. Approximately one month after MRI, a right-sided L4 hemilaminectomy was performed, and histopathological examination confirmed a sequestered lumbar disc fragment without evidence of malignancy.

The patient was pain-free at discharge, and his radicular symptoms had completely resolved. At the three-month outpatient follow-up, he remained symptom-free, and no follow-up MRI was considered clinically indicated. This case highlights the importance of considering sequestered disc fragments in the differential diagnosis of contrast-enhancing extradural spinal lesions, even when the lesion is not located in an atypical epidural compartment. When imaging findings remain inconclusive, surgical exploration with histopathological confirmation may be required to establish the correct diagnosis and guide appropriate management.

## Introduction

Lumbar disc herniation is one of the most common causes of low back pain and lumbar radiculopathy. Sequestered disc fragments represent a distinct subtype in which displaced disc material loses continuity with the parent intervertebral disc and migrates within the epidural space [1]. Although most migrated fragments remain anterior, lateral, cranial, or caudal to the affected disc level, posterior or posterolateral migration is uncommon because of the anatomical constraints of the posterior longitudinal ligament and surrounding epidural structures [2,3].

Magnetic resonance imaging (MRI) is the imaging modality of choice for evaluating intraspinal lesions. However, sequestered disc fragments may demonstrate atypical signal characteristics and peripheral rim enhancement due to inflammatory granulation tissue and neovascularization, thereby closely mimicking extradural spinal tumors, epidural abscesses, hematomas, or vascular lesions [4-7]. This diagnostic challenge is particularly relevant in patients with a history of malignancy, in whom enhancing spinal lesions are often initially suspected to represent metastatic disease.

We report a rare case of a laterally migrated sequestered lumbar disc fragment at the L4/L5 level that radiologically mimicked an enhancing extradural spinal tumor in a patient with previous colorectal and prostate carcinomas. This case highlights the diagnostic pitfalls of atypical disc migration and emphasizes the importance of histopathological confirmation when imaging findings remain inconclusive.

## Case presentation

A 75-year-old man presented with a two-month history of low back pain radiating to the right lower extremity in an L5 dermatomal distribution. He also reported intermittent paresthesia and hyperesthesia but denied bladder dysfunction, saddle anesthesia, or impairment of sexual function. There was no history of trauma or recent injury. His past medical history was significant for treated sigmoid carcinoma and prostate carcinoma.

Neurological examination revealed no objective motor deficit, with muscle strength of 5/5 in the lower extremities. The right straight-leg-raise (Lasègue) test was positive. The right patellar reflex was mildly brisk compared with the contralateral side. No relevant objective sensory deficit was identified despite the patient's intermittent sensory symptoms.

Magnetic resonance imaging (MRI) of the lumbar spine demonstrated an irregularly shaped, right-sided lateral extradural lesion at the L4/L5 level, measuring approximately 29 mm craniocaudally, 13 mm transversely, and 10 mm in the anteroposterior dimension. On axial imaging, the lesion was located lateral to the thecal sac and maintained contact with both the anterior and posterior epidural spaces. The lesion appeared isointense on T1-weighted images and hyperintense on T2-weighted sequences, with peripheral rim enhancement following contrast administration. Degenerative changes of the lumbar spine were also present, including loss of intervertebral disc height and facet arthropathy (Figures 1-5).

**Figure 1:**
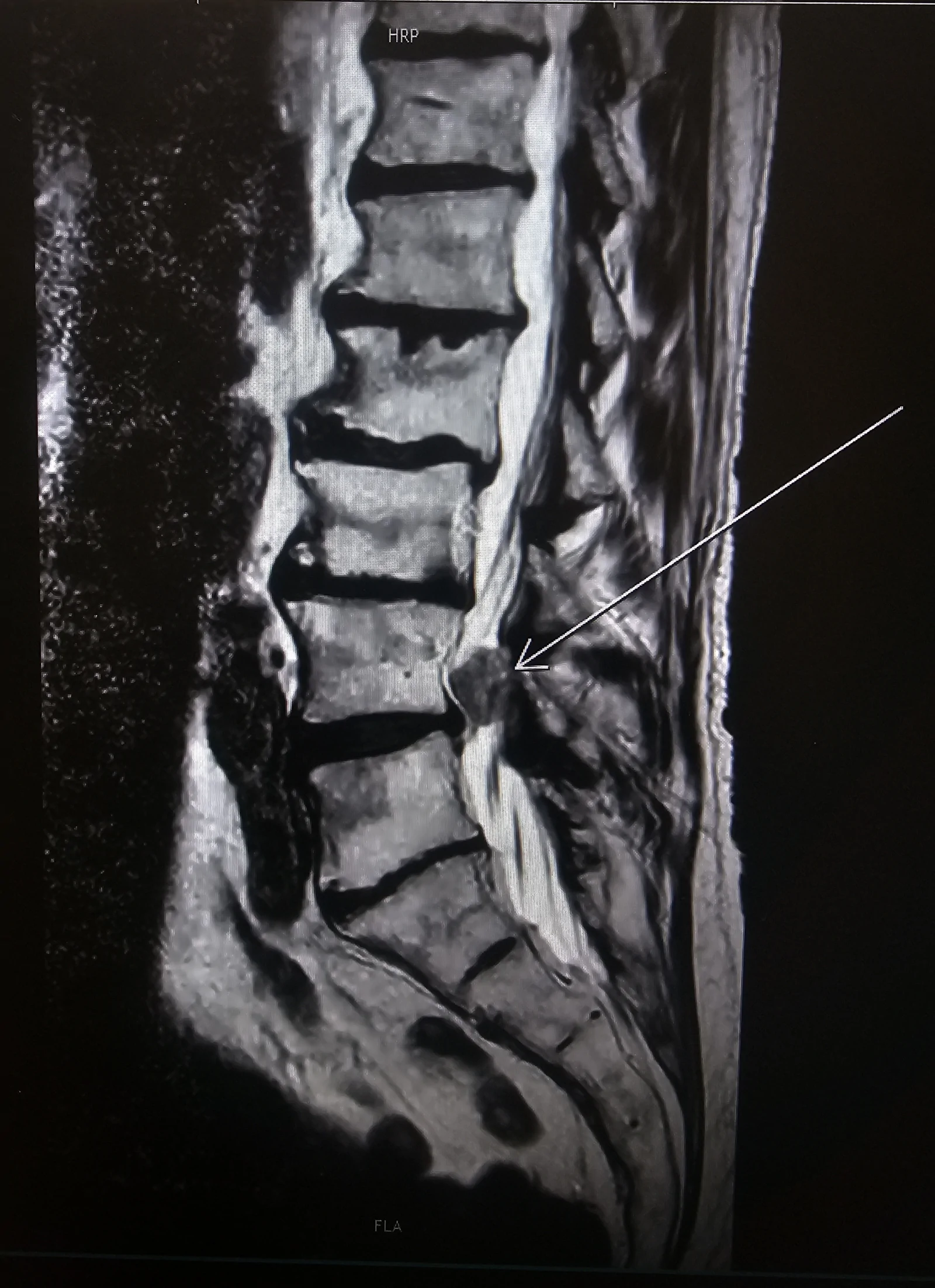
Sagittal T2 MRI Sagittal T2-weighted MRI showing a well-defined, spinal lesion at L4/5.

**Figure 2:**
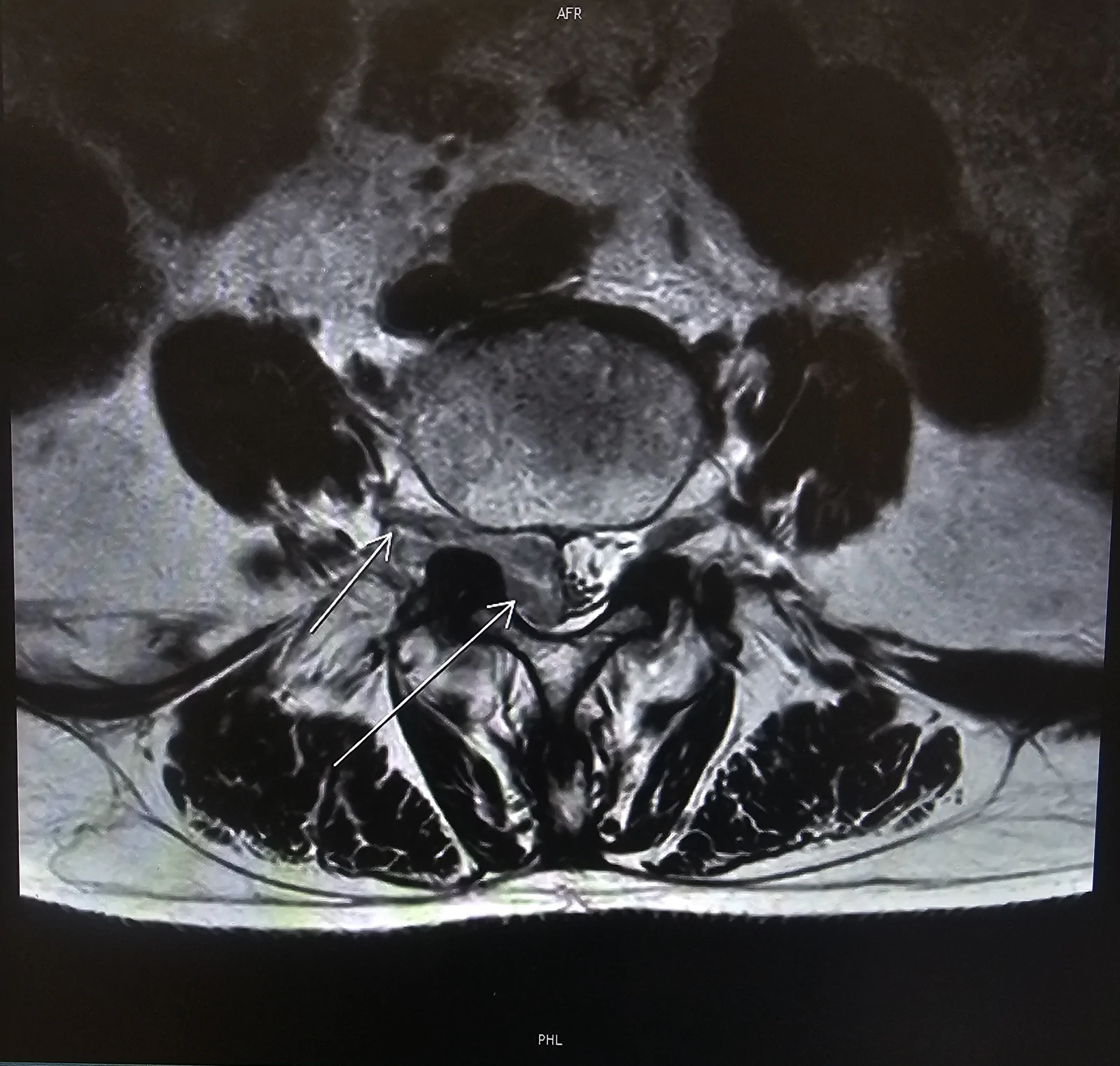
Axial T2 MRI Axial T2-weighted MRI demonstrating the right-sided lateral extradural lesion adjacent to the thecal sac. The lesion maintains contact with both the anterior and posterior epidural spaces (arrow).

**Figure 3:**
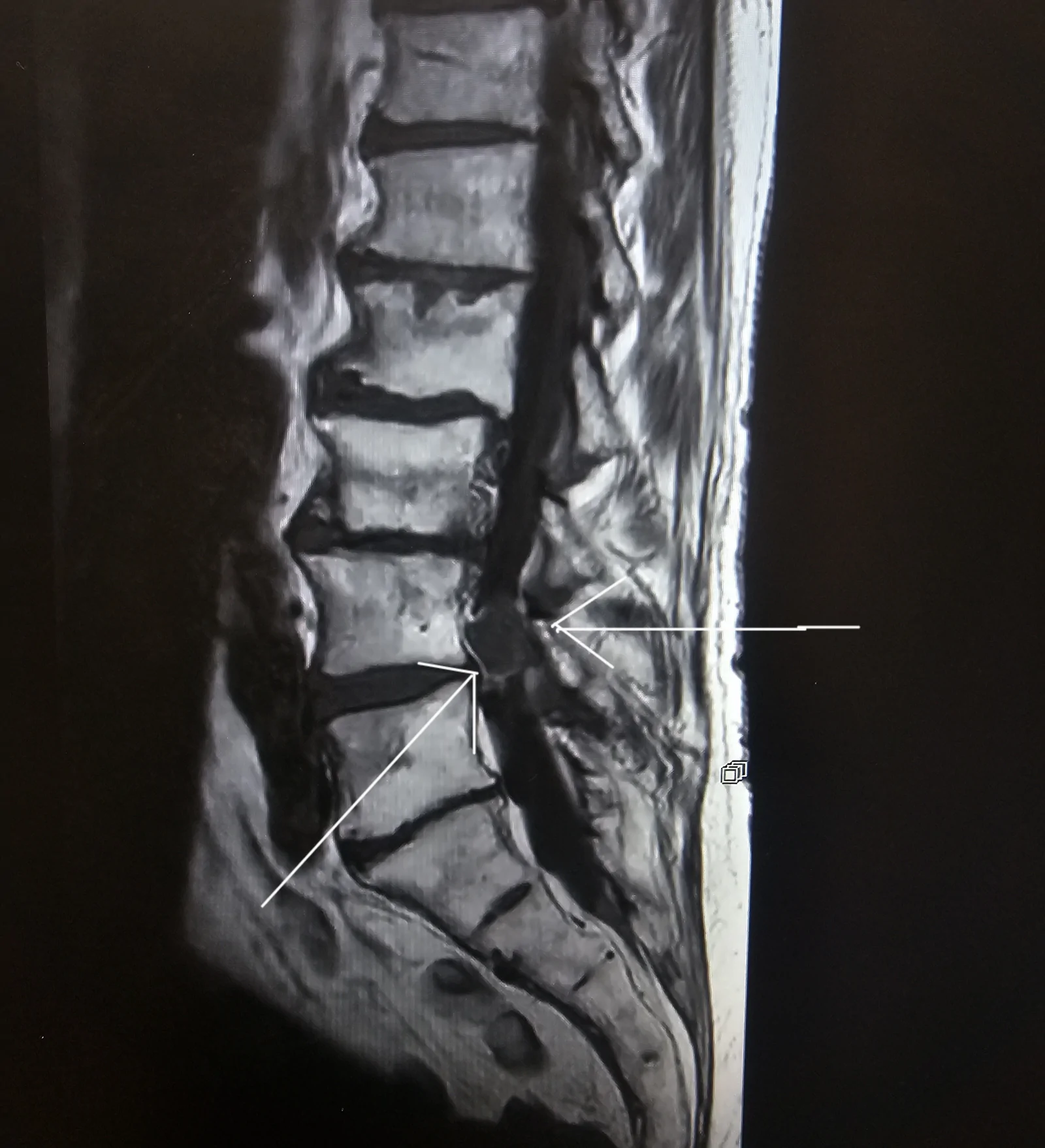
Sagittal T1 MRI Sagittal T1-weighted MRI showing rim enhancement of the lesion.

**Figure 4:**
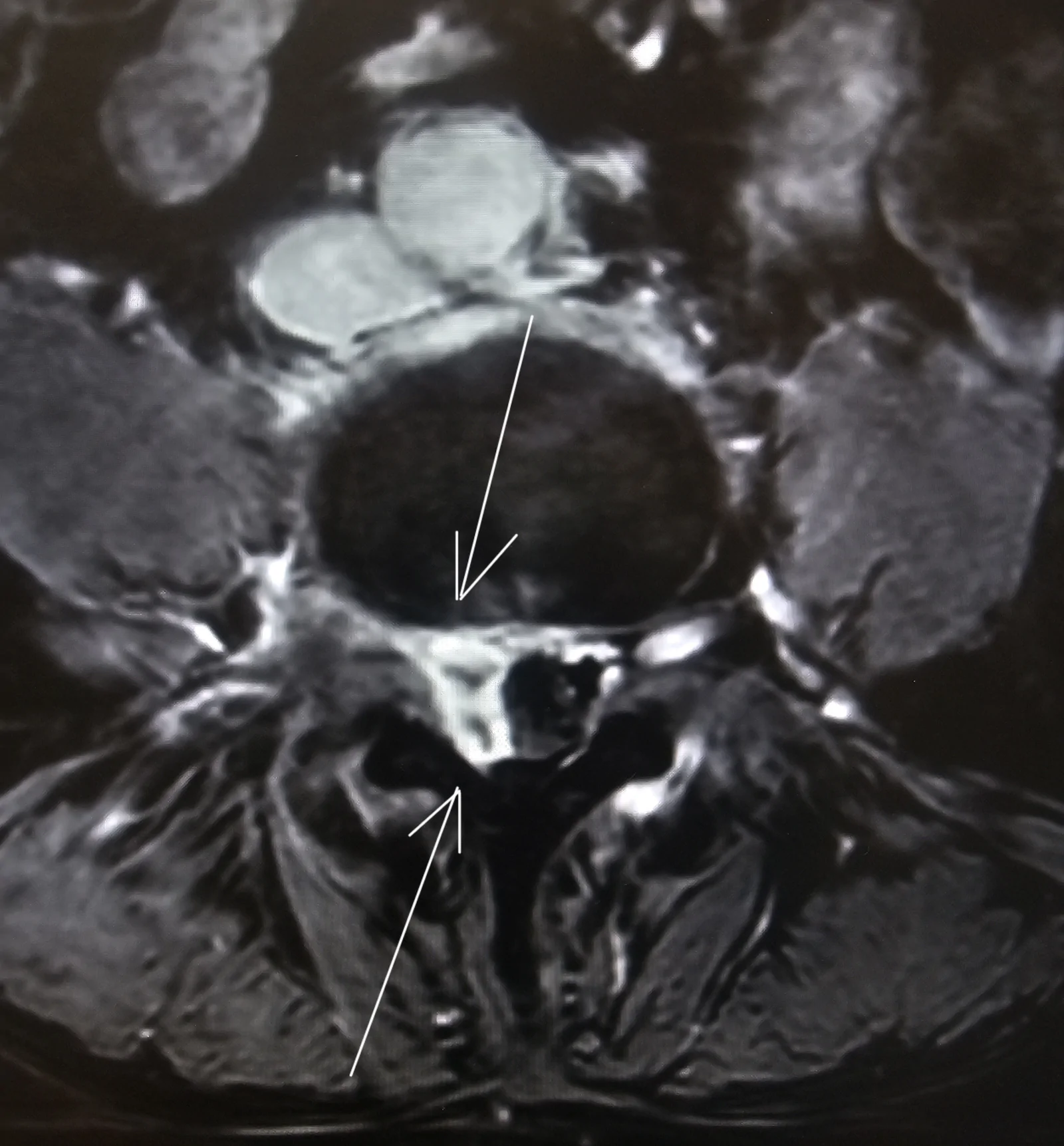
Axial post-contrast MRI Axial post-contrast T1-weighted MRI demonstrating peripheral rim enhancement of the right-sided lateral extradural lesion with compression of the thecal sac (arrow).

**Figure 5:**
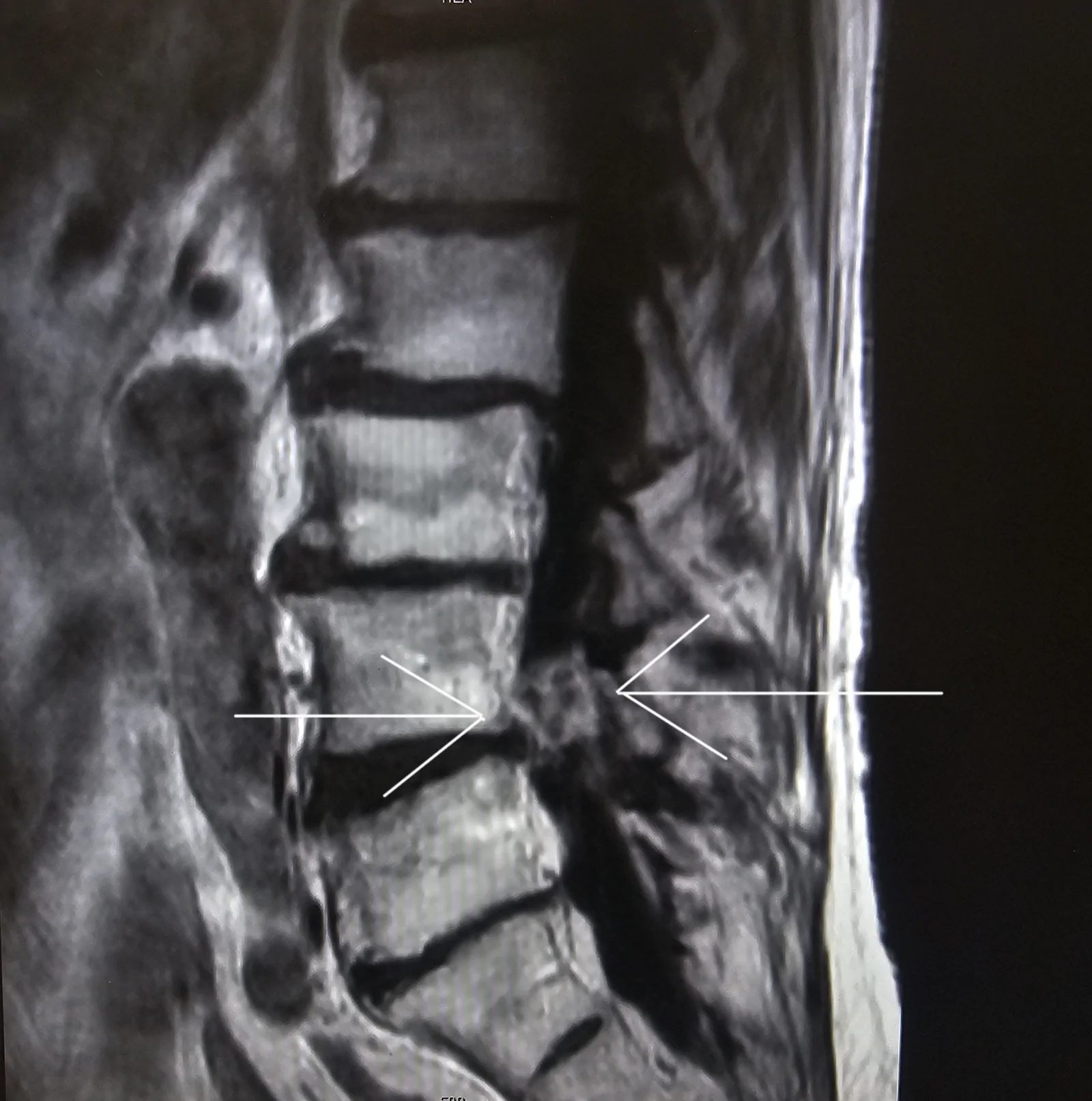
Sagittal T1 post-contrast MRI Sagittal T1-weighted post-contrast image with rim-enhancing lesion at L4/5.

Given the patient's history of malignancy and the radiological appearance, an extradural spinal tumor was considered the primary preoperative diagnosis.

Approximately one month after the MRI examination, the patient underwent a right-sided L4 hemilaminectomy. Intraoperatively, a firm extradural mass was identified lateral to the thecal sac and was densely adherent to the dural surface (Figures 6, 7).

**Figure 6:**
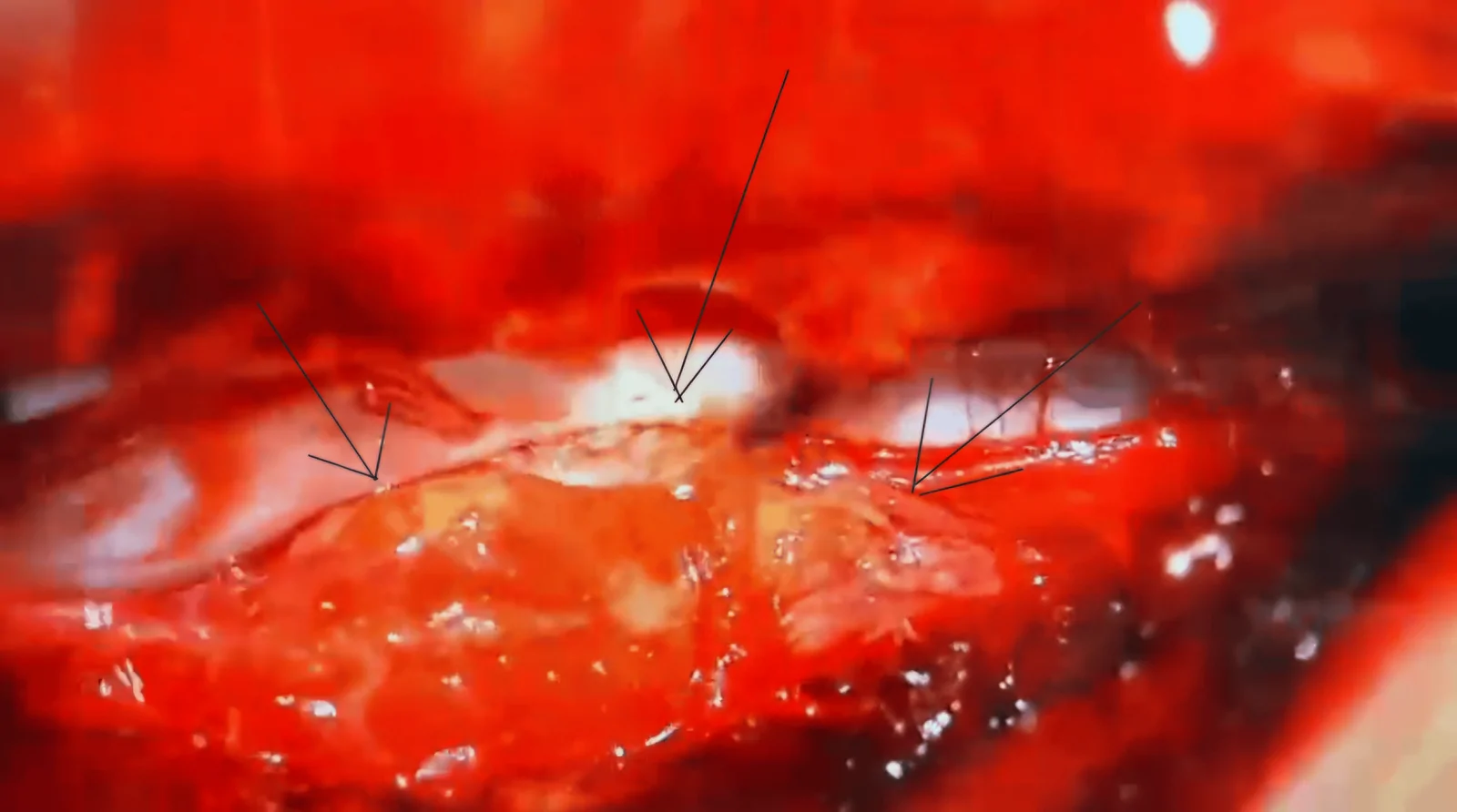
Intraoperativ microscpic image Intraoperative microscopic screenshots showing the lesion on the lateral wall of the dura with full adhesion.

**Figure 7:**
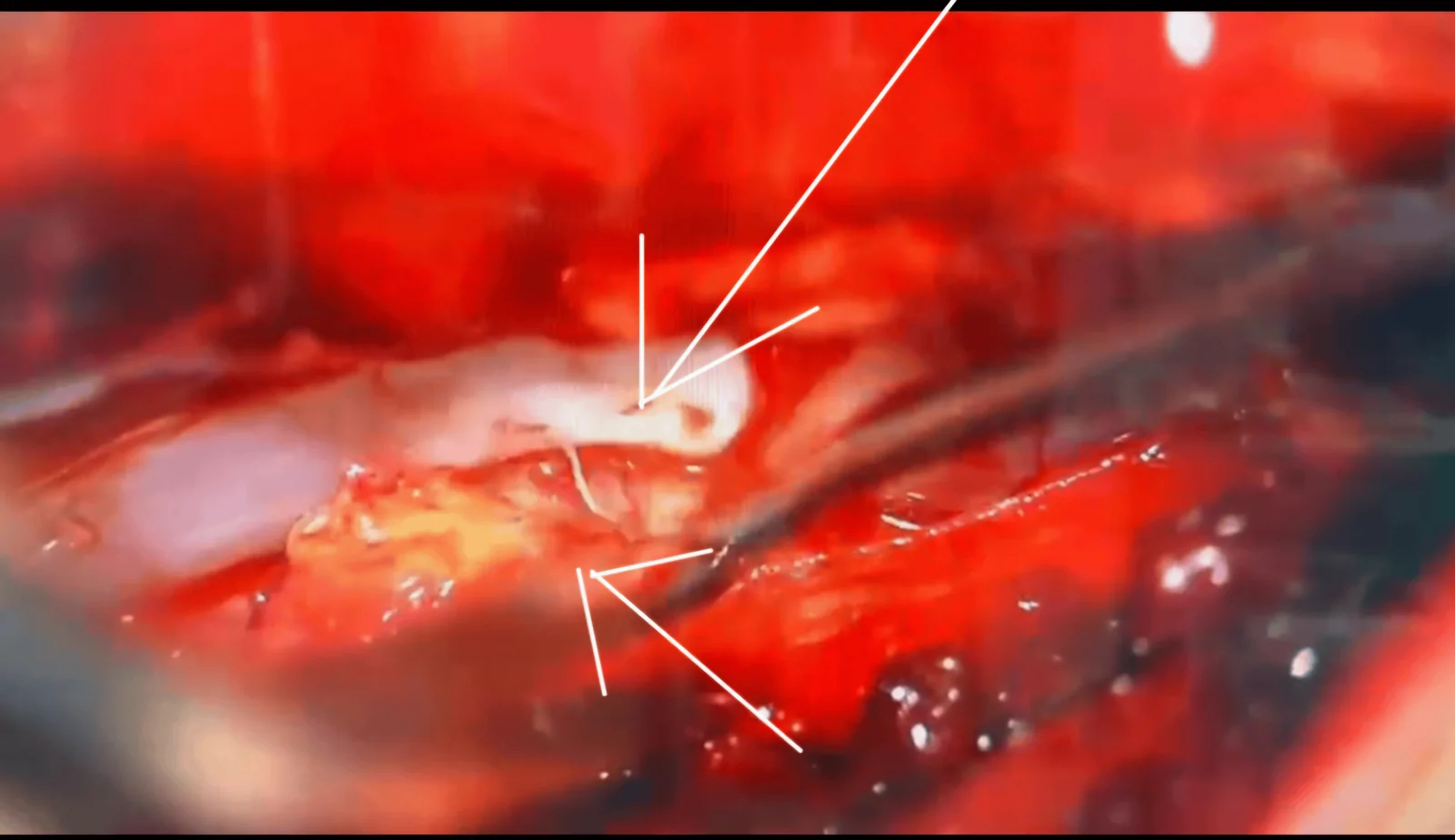
Intraoperative microscopic image Intraoperative microscopic screenshots showing the lesion on the lateral wall of the dura with full adhesion.

The lesion was completely excised without durotomy or intraoperative complications.

Histopathological examination revealed hypocellular fibrocartilaginous tissue with marked degenerative changes and focal nonspecific chronic granulation tissue, consistent with a sequestered intervertebral disc fragment. No evidence of malignancy was identified. Immunohistochemical staining for cytokeratins, prostate-specific antigen (PSA), prostate-specific membrane antigen (PSMA), and NKX3.1 was negative, excluding metastatic carcinoma (Figure 8).

**Figure 8:**
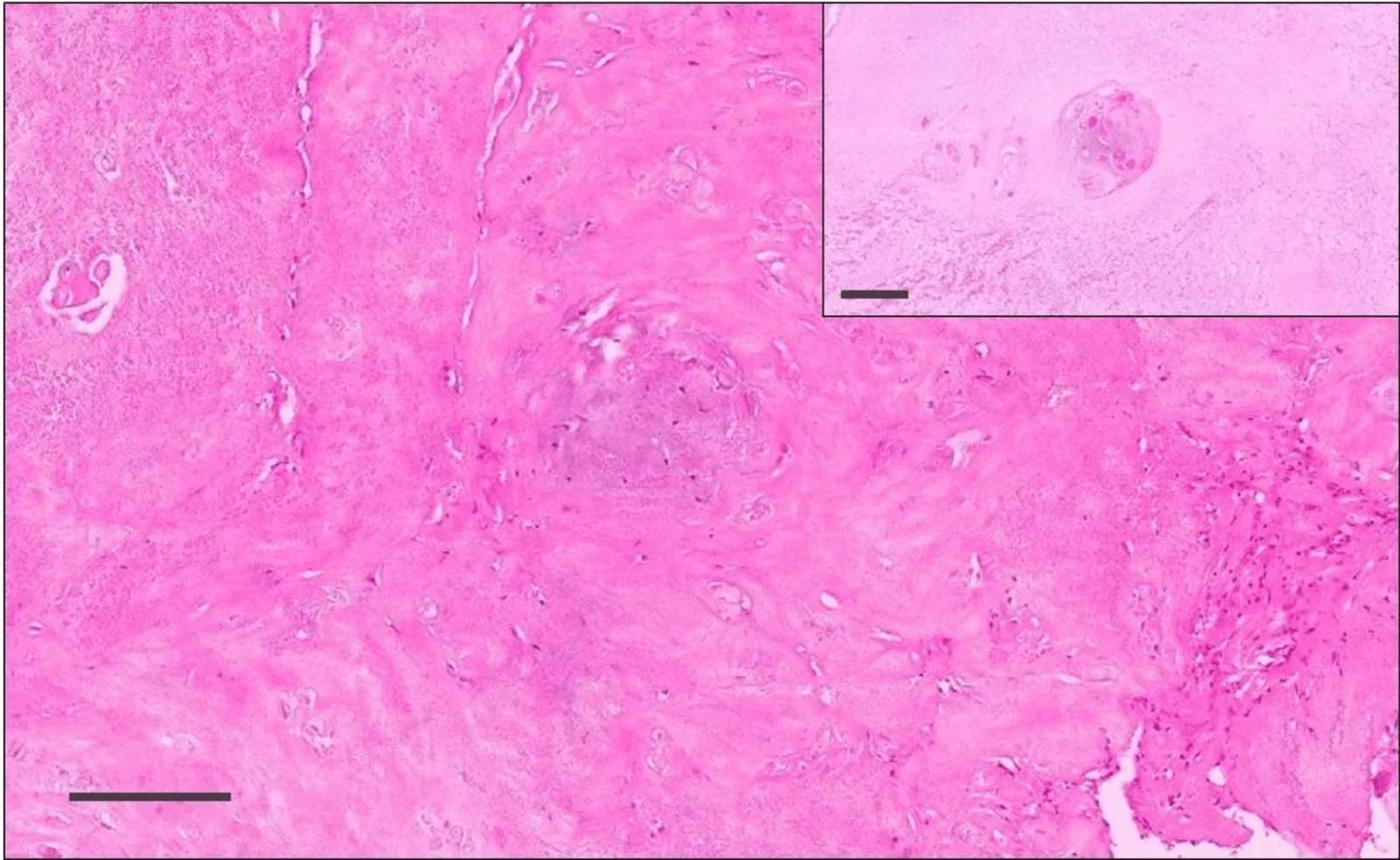
Histology of tissue sections Histology of tissue sections stained with hematoxylin and eosin showed hypocellular fibrous cartilage tissue with marked degenerative changes of the matrix, circumscribed areas of cartilage tissue necrosis (upper left corner), and focal non-specific granulation tissue (lower right corner). Note that epithelial tumor cells are absent. The scale bar corresponds to 200 µm. Insert: chondrocytes showing signs of reactive hyperplasia. The scale bar corresponds to 50 µm.

Postoperatively, the patient's radicular pain and sensory symptoms completely resolved, and he was pain-free at discharge. Motor strength remained 5/5, and the straight-leg-raise test was negative. At the three-month outpatient follow-up, the patient remained symptom-free. In the absence of recurrent symptoms or neurological deficits, additional follow-up MRI was not considered clinically indicated.

## Discussion

Sequestered lumbar disc herniation is a well-recognized cause of radiculopathy and spinal canal compromise. It is defined as displacement of disc material beyond the intervertebral disc space with complete separation from the parent disc [1]. In most cases, migrated disc fragments remain within the anterior epidural space and migrate cranially, caudally, or laterally, constrained by the posterior longitudinal ligament and surrounding epidural membranes [2,3]. Lateral or posterolateral migration is uncommon and may result in atypical clinical and radiological presentations [8-10].

Magnetic resonance imaging (MRI) is the imaging modality of choice for evaluating intraspinal lesions. However, sequestered disc fragments may demonstrate atypical signal characteristics and peripheral contrast enhancement secondary to inflammatory granulation tissue and neovascularization [4-6]. As demonstrated in the present case, peripheral rim enhancement is a recognized but nonspecific imaging feature that may also be observed in extradural tumors, epidural abscesses, hematomas, and vascular lesions such as epidural varices [7]. Consequently, MRI findings alone may be insufficient to establish a definitive diagnosis.

Several reports have described sequestered disc fragments mimicking spinal tumors, particularly when located in the posterior epidural space or when associated with contrast enhancement [8-11]. These reports emphasize the limitations of MRI in differentiating migrated disc material from neoplastic or infectious lesions. Although certain imaging findings-including the absence of osseous destruction, degenerative changes of the adjacent disc space, or subtle continuity between the lesion and the parent intervertebral disc-may favor the diagnosis of disc herniation, these features are not consistently present [4,5].

Although surgical exploration is frequently required when the diagnosis remains uncertain, the clinical relevance of this entity extends beyond the operative intervention itself. In patients with a history of malignancy, contrast-enhancing extradural lesions are commonly interpreted as metastatic disease, potentially resulting in unnecessary oncological investigations, delays in definitive treatment, or altered surgical planning. Recognizing migrated sequestered disc fragments as an important differential diagnosis may therefore facilitate more efficient clinical decision-making and appropriate patient counseling.

In the present case, the combination of a contrast-enhancing lateral extradural lesion, peripheral rim enhancement, and a previous history of colorectal and prostate carcinomas strongly favored the preoperative diagnosis of an extradural spinal tumor. On retrospective review, the lesion was located lateral to the thecal sac and maintained contact with both the anterior and posterior epidural spaces. Therefore, the diagnostic challenge in this case was not primarily attributable to an unusual site of migration, but rather to the tumor-like contrast enhancement in the setting of a significant oncological history. Surgical exploration was therefore indicated to achieve both neural decompression and definitive tissue diagnosis. Intraoperatively, the lesion was found to be extradural and densely adherent to the dura mater. Histopathological examination confirmed degenerative fibrocartilaginous disc tissue without evidence of malignancy, consistent with previously reported cases [8,10,11].

Based on the current case and the available literature, we propose a practical diagnostic approach for patients presenting with contrast-enhancing extradural spinal lesions. Initial assessment should include careful evaluation of lesion location, signal characteristics, and its relationship to the adjacent intervertebral disc. In patients with atypical imaging findings or a history of malignancy, both neoplastic and non-neoplastic etiologies should be considered. When imaging remains inconclusive, early surgical exploration with histopathological examination remains the most reliable method for establishing a definitive diagnosis and guiding appropriate management [9]. A simplified diagnostic algorithm is presented in Figure 9.

**Figure 9:**
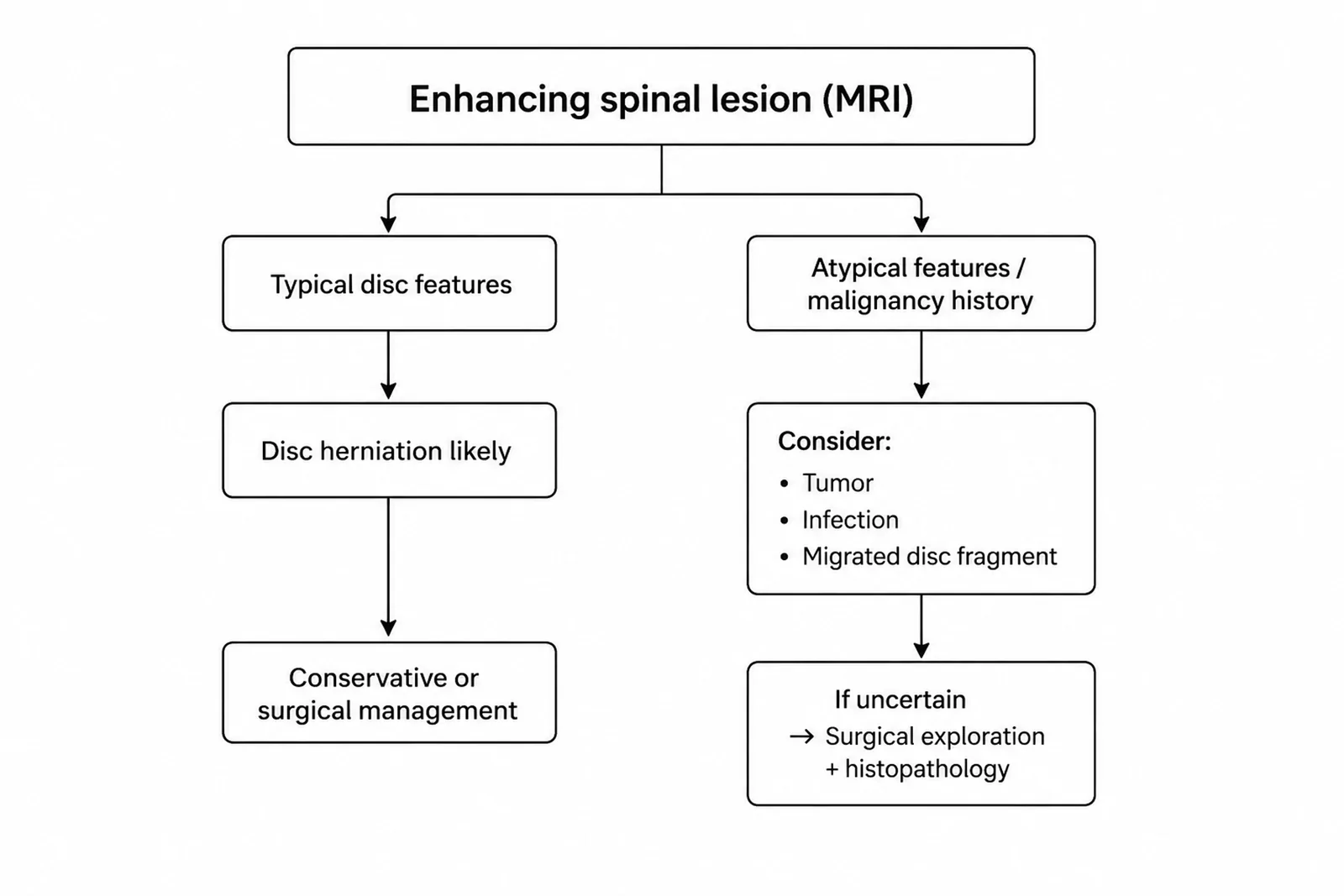
Proposed diagnostic approach Proposed diagnostic approach for contrast-enhancing extradural spinal lesions. Enhancing extradural spinal lesion identified on MRI. Initial evaluation includes assessment of imaging characteristics and clinical context. In cases with typical disc features, disc herniation is likely and managed accordingly. In atypical cases or in patients with a history of malignancy, differential diagnosis should include tumor, infection, and migrated disc fragment. When diagnosis remains uncertain, surgical exploration with histopathological confirmation is recommended.

Minimally invasive techniques, including tubular or endoscopic approaches, may be considered for both diagnostic and therapeutic purposes in carefully selected patients. However, when malignancy cannot be confidently excluded before surgery, an open surgical approach may provide superior exposure, facilitate complete lesion excision, and allow safe tissue sampling for histopathological evaluation.

In summary, sequestered lumbar disc fragments should be considered in the differential diagnosis of contrast-enhancing extradural spinal lesions, particularly in patients with a history of malignancy.

Awareness of this rare entity may reduce unnecessary diagnostic investigations, improve preoperative planning, and facilitate timely surgical management. When diagnostic uncertainty persists, surgical exploration combined with histopathological confirmation remains the gold standard for establishing the correct diagnosis.

## Conclusions

We report a rare case of a sequestered lumbar disc herniation presenting as a laterally enhancing extradural lesion mimicking a spinal tumor in a patient with a history of colorectal and prostate carcinomas. This case highlights the diagnostic challenges posed by tumor-like MRI findings and emphasizes the importance of considering sequestered disc fragments in the differential diagnosis of contrast-enhancing extradural spinal lesions. Although MRI remains the imaging modality of choice, histopathological confirmation following surgical exploration remains the gold standard when the diagnosis is uncertain. Increased awareness of this uncommon entity and a systematic diagnostic approach may facilitate timely diagnosis, appropriate surgical planning, and avoidance of unnecessary diagnostic investigations.
